# A Longitudinal ^1^H NMR-Based Metabolic Profile Analysis of Urine from Hospitalized Premature Newborns Receiving Enteral and Parenteral Nutrition

**DOI:** 10.3390/metabo12030255

**Published:** 2022-03-17

**Authors:** Nuria Esturau-Escofet, Eduardo Rodríguez de San Miguel, Marcela Vela-Amieva, Martha E. García-Aguilera, Circe C. Hernández-Espino, Luis Macias-Kauffer, Carlos López-Candiani, José J. Naveja, Isabel Ibarra-González

**Affiliations:** 1 Instituto de Química, Universidad Nacional Autónoma de México, Mexico City 04510, Mexico; esturau.nuria@gmail.com (N.E.-E.); q.martha.garcia@gmail.com (M.E.G.-A.); circas0310@gmail.com (C.C.H.-E.); navejaromero@gmail.com (J.J.N.); 2 Departamento de Química Analítica, Facultad de Química, Universidad Nacional Autónoma de México, Mexico City 04510, Mexico; erdsmg@unam.mx; 3 Laboratorio de Errores Innatos del Metabolismo y Tamiz, Instituto Nacional de Pediatría, Secretaría de Salud, Mexico City 04530, Mexico; mvelaa@pediatria.gob.mx; 4 Unidad de Genómica de Poblaciones Aplicada a la Salud, Facultad de Química, Universidad Nacional Autónoma de México/Instituto Nacional de Medicina Genómica, Secretaría de Salud, Mexico City 04809, Mexico; luisrmacias@gmail.com; 5 Departamento de Neonatología, Instituto Nacional de Pediatría, Secretaría de Salud, Mexico City 04530, Mexico; clopezcandiani@gmail.com; 6 Unidad de Genética de la Nutrición, Instituto de Investigaciones Biomédicas, Universidad Nacional Autónoma de México/Instituto Nacional de Pediatría, Secretaría de Salud, Mexico City 04530, Mexico

**Keywords:** prematurity, enteral feeding, parenteral nutrition, ^1^H NMR-based metabolic profile, chemometric analysis

## Abstract

Preterm newborns are extremely vulnerable to morbidities, complications, and death. Preterm birth is a global public health problem due to its socioeconomic burden. Nurturing preterm newborns is a critical medical issue because they have limited nutrient stores and it is difficult to establish enteral feeding, which leads to inadequate growth frequently associated with poor neurodevelopmental outcomes. Parenteral nutrition (PN) provides nutrients to preterm newborns, but its biochemical effects are not completely known. To study the effect of PN treatment on preterm newborns, an untargeted metabolomic ^1^H nuclear magnetic resonance (NMR) assay was performed on 107 urine samples from 34 hospitalized patients. Multivariate data (Principal Component Analysis, PCA, Orthogonal partial least squares discriminant analysis OPLS-DA, parallel factor analysis PARAFAC-2) and univariate analyses were used to identify the association of specific spectral data with different nutritional types (NTs) and gestational ages. Our results revealed changes in the metabolic profile related to the NT, with the tricarboxylic acid cycle and galactose metabolic pathways being the most impacted pathways. Low citrate and succinate levels, despite higher glucose relative urinary concentrations, seem to constitute the metabolic profile found in the studied critically ill preterm newborns who received PN, indicating an energetic dysfunction that must be taken into account for better nutritional management.

## 1. Introduction

Preterm birth is defined as any birth before 37 completed weeks of gestation. Preterm infants are extremely vulnerable to a range of morbidities, complications, and death [1]. The World Health Organization (WHO) has estimated that preterm birth causes one million neonatal deaths annually [2], accounting for 75% of perinatal mortality and more than 50% of long-term infant morbidity; moreover, complications related to prematurity may require medical treatments and frequent hospitalizations [3]. Preterm birth is a serious global problem due to its socioeconomic burden [1,4]. One critical management issue in preterm newborns is their nutrition because they have limited nutrient stores at birth and it is difficult to establish enteral feeding, which leads to inadequate growth, frequently associated with poor neurodevelopmental outcomes [5,6]. A recent position paper of the European Society for Paediatric Gastroenterology, Hepatology and Nutrition Committee on Nutrition (ESPGHAN) declared that the nutritional management of critically ill preterm neonates varies widely and that there are controversies regarding the time and mode of feeding. Research on nutritional support in critically ill preterm infants is needed to resolve uncertainties regarding metabolism in this vulnerable population [7]. Although parenteral nutrition (PN) is widely used, there are still questions about the best biochemical parameters to define its benefits and potential risks to optimize its composition [8,9,10,11]. Biochemical classical blood studies in preterm newborns are difficult due to their fragility, low weight, and immaturity; therefore, it is important to use other biological matrices, such as urine, whose collection is minimally invasive. Metabolomics is defined as the analysis of low-molecular-mass metabolites involving substrates or products of the biochemical pathways of living systems. Mass spectrometry (MS) and nuclear magnetic resonance (NMR) spectroscopy are the most commonly used analytical techniques in metabolomics [12]. Clinical metabolomics is a tool used to measure the biochemical adaptations of organisms to diseases, with the intention of finding characteristic metabolic profiles or signatures that could be used as biomarkers [13]. Metabolomic analysis of biofluids could be used to investigate metabolic changes in preterm newborns with different pathologic conditions, such as respiratory depression, malformations, jaundice, acute kidney injury, necrotizing enterocolitis, and intraventricular hemorrhage [3,12,13,14,15,16,17,18,19,20]. Urinary metabolomic analysis is especially valuable for studying this vulnerable population. Moreover, urine, unlike blood, lacks mechanisms for maintaining homeostasis; therefore, it is an ideal source of biomarkers that can reflect systemic biochemical changes [21,22]. The aim of this study was to use untargeted ^1^H NMR to analyze urine samples from hospitalized preterm newborns with critically ill conditions who received PN or enteral nutrition (EN) to determine whether there are metabolomic differences according to nutritional type (NT).

## 2. Results

### 2.1. Patients and Samples

**Figure 1:**
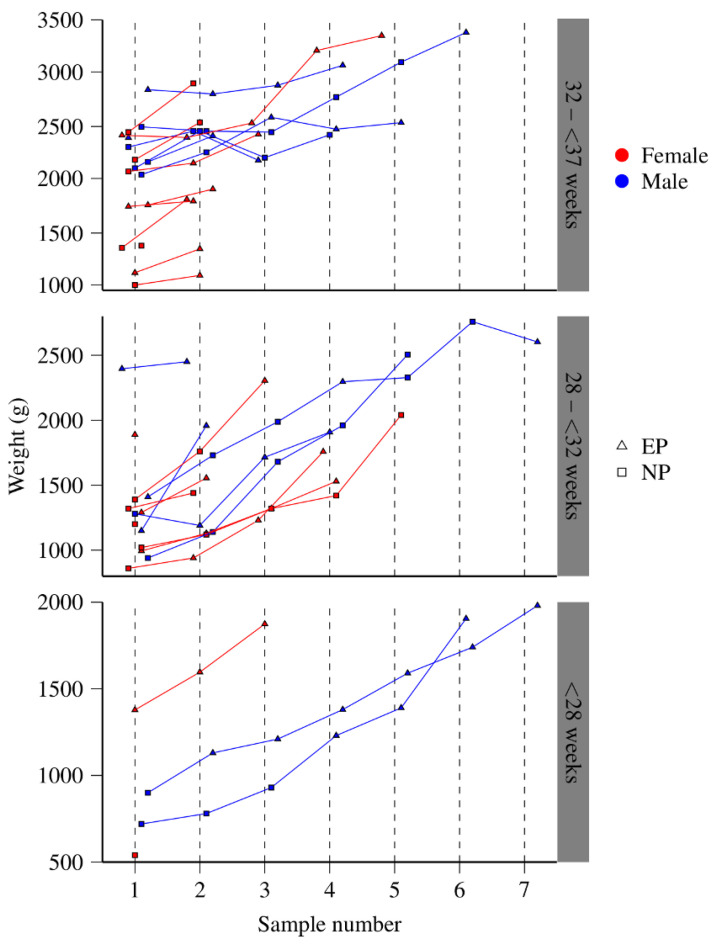
Number of samples and weight at the time of each sampling of the studied preterm newborns, grouped by gestational age, sex, and birth weight (107 urine samples from 34 preterm newborns).

A total of 34 preterm newborns (20 girls and 14 boys) hospitalized in the neonatal intensive care unit (NICU) were included in the study. The average gestational age was 31 weeks, with a range from 26 to 36 weeks, and the mean birth weight was 1.587 kg (740–2945 g). The mean height at birth was 40.5 cm (32 to 48 cm). Twenty-two out of 34 (64.7%) newborns had adequate weight for gestational age, and 12/34 (35.29%) had small weight for gestational age. The average Apgar score at minute 1 was 7 (min. 2 to max. 9), and at 5 min, it was 8 (min. 5 to max. 9). At the initial sampling, the average age of the preterm newborns was 12 days of life, with a mean weight of 1.601 kg (540 to 2840 g) and a mean height of 41 cm (31.5 to 51 cm). The number of samples and the weight at the time of each sampling of the studied preterm newborns are shown in Figure 1. The main causes of admission to the NICU are summarized in Table 1. The shortest hospital stay was one day, and the longest was 70 days (average: 33 days). All patients were discharged home except two who died: one of them, a female preterm newborn of 26.5 weeks of gestation, due to hyaline membrane disease, necrotizing enterocolitis, sepsis, disseminated intravascular coagulation, and pulmonary hemorrhage; and the second one, a female preterm newborn of 29 weeks of gestation, due to hyaline membrane disease, sepsis, and hemorrhagic shock. One hundred and seven urine samples, 48 from those who received PN and 59 from those who received EN, were collected periodically during hospitalization and were analyzed. The ^1^H NMR spectra of these samples were further employed for principal component analysis (PCA) and partial least squares discriminant analysis (PLS-DA).

### 2.2. Metabolomic Profile Analysis by PCA and OPLS-DA

**Figure 2:**
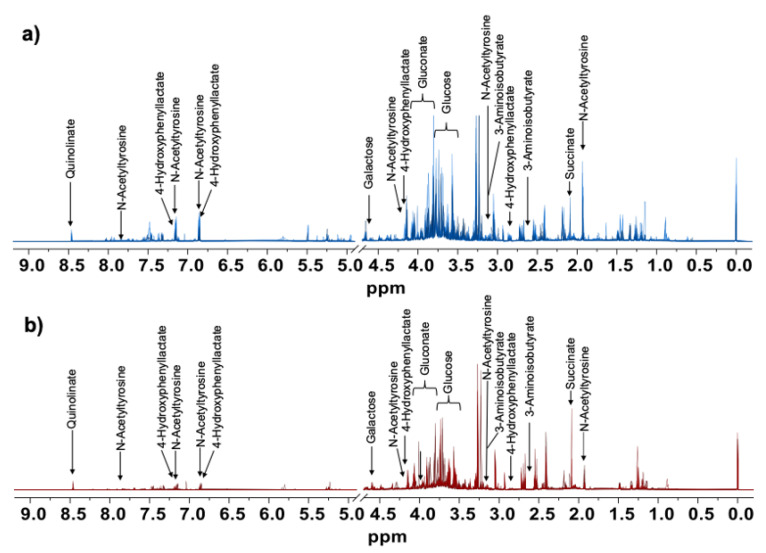
Overlay of 12 stacked ^1^H NMR spectra (700 MHz, 298 K, D_2_O (KH_2_PO_4_, pH 7.4) normalized to the total area of the urine samples of preterm newborns who received (**a**) PN or (**b**) EN. Some metabolites are shown on top of each spectrum.

Figure 2 shows an overlay of 12 stacked urine ^1^H NMR spectra covering the range δ −0.5–9.0 ppm with representative metabolites assigned for preterm newborns who received PN (Figure 2a) or EN (Figure 2b). The metabolites were assigned according to the chemical shifts and coupling constants.

**Figure 3:**
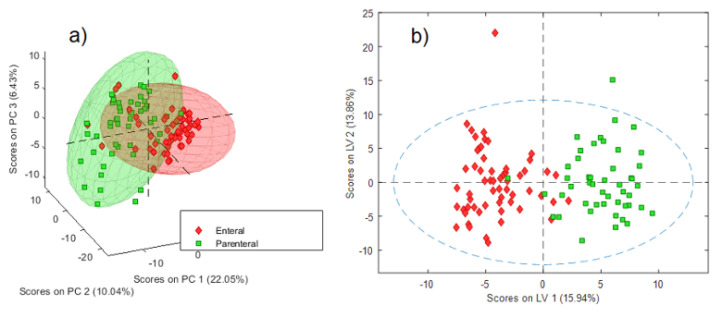
(**a**) PCA score plot for the first three components of a 6-component model of the ^1^H NMR spectra showing partial differentiation between groups with different NTs. (**b**) OPLS-DA score plot for a 4-component model of the ^1^H NMR spectra showing good differentiation between groups with different NTs.

The ^1^H-NMR spectra were subjected to exploratory PCA. The scree plot of the model did not show a sharp reduction in the root mean square error of calibration (RMSEC) and root mean square error of cross-validation (RMSECV) values; however, based on the second parameter, a six-component model was evaluated. This model explained 52.74% of the spectral variability (22.05, 10.04, 6.43, 5.63, 4.63, and 3.96% of variance explained by each component). No anomalous data were identified in the Hotelling T_2_ reduced vs. Q residuals reduced plot at the 95% confidence interval. Among the different data characteristics used to screen the profiles, i.e., sex, weight, height, and NT (namely, PN or EN), only NT was related to some degree of differentiation between groups in the score plot, as shown in Figure 3a. Supervised modeling by orthogonal partial least squares discriminant analysis (OPLS-DA) was further performed using NT as the response variable. The scree plot revealed four latent variables based on a CV classification error of 13.87% and an R2Y of 70.18%. Good performance of the model was observed (Q2 52.59%), as denoted by the separation between both groups along the first latent variable (Figure 3b).

To assess the significance of the OPLS-DA model, a 300 random permutation test was performed, showing that the calculated R^2^ (0.0, 0.318) and Q^2^ (0.0, −0.374) interval variation values were far lower than the associated model values, 0.7018 and 0.5259, respectively (Supplementary Figure S1). This finding indicates that the classification is significant and not attributable to random data variation, i.e., spurious relationships.

In addition, the AUC values of the ROC curve in calibration and cross-validation reached 0.9908 and 0.9216, respectively, indicating good discriminant power. Based on the variable importance in the projection (VIP) values (VIP > 1.5), the chemical shifts associated with discrimination between the groups were selected and identified. Table 2 shows the identified metabolites and their Human Metabolome Database (HMDB) identification (ID)

**Figure 4:**
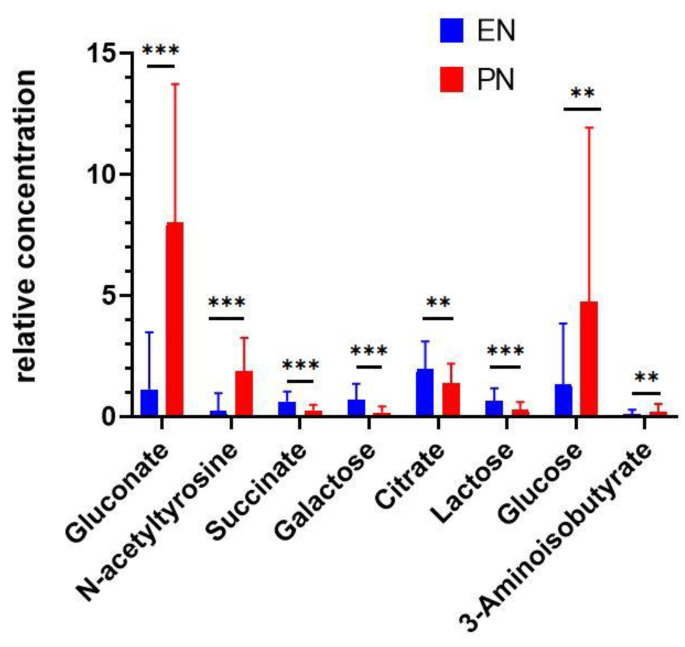
Univariate analyses of relevant metabolites producing significant differences between NT groups identified according to the S-line plot. EN = enteral nutrition; PN = parenteral nutrition. ** *p* < 0.01, *** *p* < 0.001.

Using the S-line plot, further selection of the NMR shifts that enhanced the classification was performed (Supplementary Figure S2). Univariate analyses of the relative concentrations of each metabolite in Table 2 were additionally performed to verify the significance of the results by a two-group independent samples comparison t test. The *p* values obtained are shown in Table 2. Interestingly, mainly metabolites with higher absolute values of the correlation loading in the S-line plot (>0.6) gave significant differences (95% confidence level), except for 4-hydroxyphenyllactate. This result is also illustrated in Figure 4, where a graphical representation of the univariate analyses with the significant metabolites is shown. Clearly, in the case of 4-hydroxyphenyllactate, its very low concentration in both groups (EN and PN) in comparison to the other metabolites may be a plausible cause of the nonsignificant effect despite having a high correlation loading. 3-Aminoisobutyrate barely gave a significant result (*p* = 0.048820), having a correlation loading close to 0.5, which may be indicative of a compromising limiting case between the value of the loading and the very low concentration.

### 2.3. Interpretation of the Involved Metabolic Pathways

**Figure 5:**
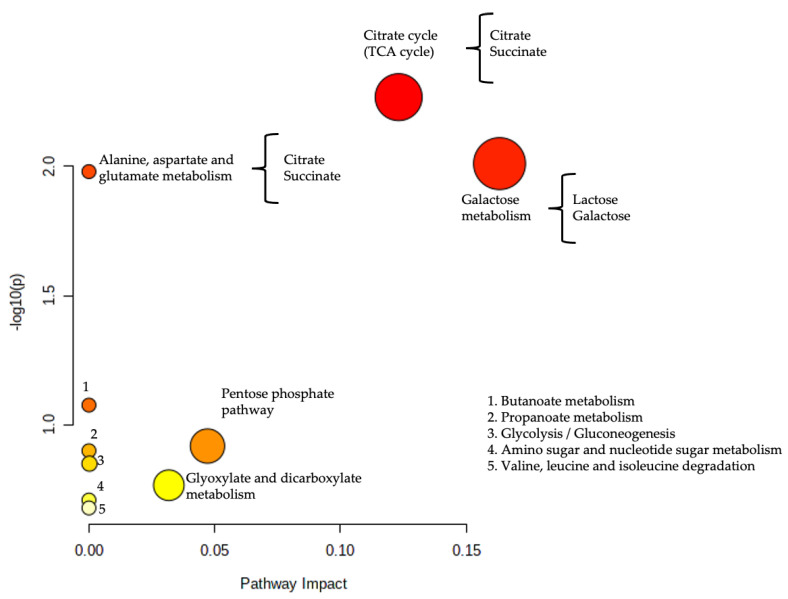
Metabolic pathway alterations observed in the studied preterm newborns. The x-axis represents the topology analysis (pathway impact), and the y-axis represents the enrichment analysis (−log(*p*)). The color and size of each pathway were set according to the *p* values and pathway impact values, respectively.

Metabolic pathway analysis (Figure 5) showed 10 altered biochemical pathways; two of them were identified as significantly enriched (*p* value < 0.05; y-axis), and the galactose and citrate cycle pathways had the larger impact score (>0.1; x-axis). The detailed results from the pathway analyses are shown in Supplementary Table S1.

### 2.4. Metabolomic Profiles by Parallel Factor Analysis (PARAFAC-2)

**Figure 6:**
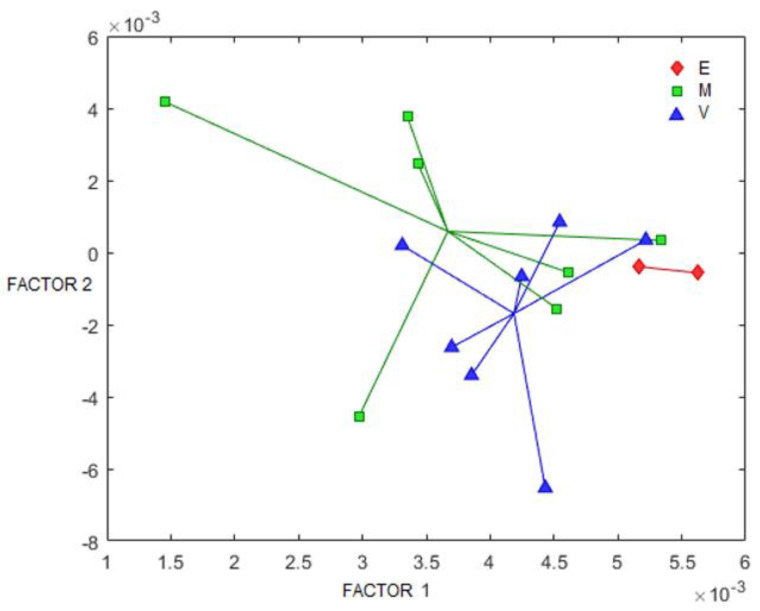
Loading plot (factor 1 vs. factor 2) of the third mode (subjects) in the PARAFAC-2 model. Labels correspond to the gestational age at birth group E = extremely preterm (less than 28 weeks of gestation); V = very preterm (28–32 weeks); and M = moderate to late preterm (32–37 weeks).

As only some (39.65%) of the variation in the X-block could be clearly associated with NT in the urine samples and the models above do not consider the longitudinal characteristic of the study, PARAFAC-2 was employed as an additional chemometric method to isolate possible sources of data variability. This chemometric method allowed us to analyze 3-mode data or components by isolating the common contribution of two of the dimensions (^1^H NMR spectra and subjects) from the contributions of the first dimension (time) taken separately for each subject, as, ultimately, each subject was measured in a distinct time. For the analysis, mode 1 corresponded to time (sampling day); mode 2 corresponded to ^1^H NMR spectra; and mode 3 corresponded to the subjects. A total of 17 of the 32 patients were selected to guarantee that at least three observations were made for each subject, i.e., subjects with only one or two observations were excluded. The results showed that the percent of variance captured by a two-factor model reached 63.30 and 26.29% in the X-block, which is greater than previous values in PCA and OPLS-DA, as expected by considering the longitudinal character of the study. These two factors were retained according to the 100% core consistency achieved (Supplementary Figure S3). Additionally, a further increase in the number of factors tended to reduce core consistency and to augment the deviations between the observed and expected behavior of this parameter. The mode 1 (time) loading plot (Supplementary Figure S4) showed as many loadings as the number of components per subject’s samples (2 factors × 17 subjects), i.e., the loading values of each component at the specific times each subject was observed. Clearly, not all the subjects were assessed at the same time, and this fact was addressed by using the PARAFAC-2 algorithm. The mode 2 (^1^H NMR spectra) loading plot (Supplementary Figure S5) shows the observed spectrum pattern of the two components that are common for all subjects’ samples. The higher the difference between both components at a determined chemical shift, the more important this chemical shift is in the model. The mode 3 loading plot is shown in Figure 6. This graph is equivalent to the PCA sample scores, and each subject has characteristic sample scores that define the status in the transformed 3-dimensional space. As observed, differentiation among the groups according to the gestational age at birth was observed. The 2-mode loading plot was then used to identify the buckets’ chemical shifts associated with such distinction (Table 3).

## 3. Discussion

These results show that PN treatment induces changes in the urine of critically ill preterm newborns that can be shown through untargeted ^1^H NMR. Our results demonstrate, both in the OPLS-DA multivariate (Table 2) and univariate (Figure 4) statistical analyses, differences in the urinary concentrations of nine metabolites (gluconate, glucose, N-acetyltyrosine, citrate, lactose, succinate, galactose, and 3-aminoisobutyrate). These differences constitute a profile of exposure to PN in the studied patients. In this work, we also found some non-assigned signals (Table 2), but their significance is not known. Interestingly, the non-assigned signal at 7.68 ppm has been described in the urine of preterm newborns by Moltu et al. [15]. It would be important to identify all the non-assigned signals with other analytical methodologies to determine their implications in the metabolism of critically ill preterm newborns.

PARAFAC-2 showed that other metabolites, such as betaine, myo-inositol, creatinine, and glycine, are significantly different according to gestational age, without relation to PN treatment in the studied preterm newborns. The influence of gestational age on the urinary metabolome of the preterm newborns has been previously reported in the literature [23,24,25]. The fact that differences in the relative concentrations of glucose, succinate, gluconate, and 4-hydroxyphenyllactate were demonstrated by the two independent analyses (PARAFAC-2 and univariant) indicates a relation with the NT and gestational age.

To determine the biological meaning of the results, we performed a pathway analysis of the metabolites based on their relevance obtained in the S-line plot (Figure 4), showing alterations of 10 metabolic pathways: butanoate; propanoate metabolism; glycolysis/gluconeogenesis; amino sugar and nucleotide sugar metabolism; branched chain amino acid degradation; glyoxylate and dicarboxylate metabolism; pentose phosphate pathway; alanine/aspartate/glutamate metabolism; tricarboxylic acid cycle (TCA); and galactose metabolism. Two of these pathways (TCA and galactose metabolism) were identified as the most significantly enriched (*p* < 0.05) and had large impact scores (>0.1, Figure 5).

According to MetaboAnalyst 5.0, in the TCA cycle, a total of 20 compounds are involved (Supplementary Table S1) [26]. In this work, the impact of the TCA cycle was mainly due to differences in the relative concentrations of citrate and succinate, which were found to be lower in the patients exposed to PN. The mitochondrial TCA cycle is important in energy metabolism, providing intermediates for the synthesis of glucose and some amino acids [15]. Our results indicate that in critically ill preterm newborns who receive PN, the TCA cycle is dysregulated due to lower relative concentrations of citrate and succinate. Citric acid is an intermediate of the TCA cycle, synthesized by condensation of oxaloacetate with acetyl-CoA via the enzyme citrate synthase. Succinic acid is also an intermediate of the TCA cycle, produced from alfa-ketoglutaric acid, catalyzed by the enzyme alpha-ketoglutaric acid dehydrogenase [27], and reduced excretion of TCA cycle intermediates is indicative of impaired oxidative metabolism [28]. Low relative concentrations of succinate and citrate in urine samples have also been observed in preterm newborns with chorioamnionitis antecedents, but their nutritional status has not been reported [29]. These findings could suggest an energy deficit in the studied preterm newborns fed PN and could be useful in revising its content, especially the provided energy supply. Recent publications regarding PN in pediatric patients state that inadequate energy supply can increase the risk of serious complications, such as suboptimal motor, cognitive and behavioral development, impaired immunity, impaired growth, and death [8,30].

Notably, in our work, the dysregulation of the TCA cycle observed in the studied patients exposed to PN occurs despite the finding of significantly higher glucose levels (Figure 4), so the energetic content of PN seems to be lower than that currently recommended [8,15].

Moreover, the galactose metabolic pathway has the intervention of 27 compounds, with galactose and lactose being the main implicated metabolites (Supplementary Table S1) [26]. In this study, we found a lower relative concentration of galactose and lactose in preterm newborns exposed to PN, which could be explained since they were not receiving any of the sources that provide these metabolites, such as breast milk or formula.

One limitation of this study is that some metabolites, such as 3-aminoisobutyrate, have been found in term newborns with respiratory depression, malformations, and viral infections [13,31], so other factors other than gestational age could be involved. Furthermore, the metabolomic studies performed in preterm newborns and published to date are varied in their design and are not fully comparable, and multicentric studies on a larger number of patients are needed [32,33].

## 4. Materials and Methods

### 4.1. Ethics Statement

This study was registered and approved by the Institutional Committees for Research, Ethics and Biosecurity (INP-064/2013; INP-062/2018) in accordance with the principles of the Helsinki Declaration, and written informed consent was obtained from the parents of the participants.

### 4.2. Patients and Sampling

One hundred and seven urine samples were collected from 34 hospitalized preterm newborns who were recruited from the NICU at the National Institute of Pediatrics in Mexico City and were followed throughout their hospital stay. The general scheme of the study, with the number of samples taken from each preterm newborn and the corresponding weight, sex, and NT for each subject, is shown in Figure 1. Although in the studied population, the comorbidities were very varied, a two-way ANOVA analysis indicated a non-significant effect of those comorbidities in relation to NT (EN or PN) at the 95% confidence level (Supplementary Table S2). Approximately 1 mL of urine was collected from the studied newborns using sterile urine plastic bags at the time of admission and thereafter weekly until discharge. The minimum number of urine samples of the included patients was one, and the maximum was seven. The samples were stored at −70 °C until ^1^H NMR analysis. The samples were categorized according to their NT as follows: (a) PN and (b) EN.

Clinical data and comorbidities were recorded according to the clinical practices and standard definitions. Patients with a confirmed diagnosis of inborn error of metabolism were excluded. Prematurity classification of the patients was performed according to OMS in the following groups: extremely preterm (less than 28 weeks of gestation); very preterm (28–32 weeks); and moderate to late preterm (32–37 weeks).

### 4.3. PN or EN Used

All the preterm newborns received the same PN or EN. The characteristics of the PN are shown in Supplementary Table S3. Briefly, 4 g/kg of proteins, 17.2 g/kg of carbohydrates, and 3.5 g/kg of lipide were given. The EN was given with commercial preterm formula (Enfamil Prematuros^®^ Mead Johnson^®^, Mexico City, Mexico), 24 kcal/30 mL, maximum 180 mL/kg/day (Supplementary Table S4). A dosage of 100 to 200 mg/kg/day of 10% calcium gluconate and 50 mg/kg/day of magnesium sulfate was used.

### 4.4. ^1^H NMR Analysis

An untargeted metabolomic ^1^H NMR assay was performed on the 107 urine samples. After thawing, each urine sample (500 μL) was centrifuged at 3500 rpm for 5 min at 4 °C. Each sample (400 μL) plus 200 μL of phosphate buffer prepared in D_2_O (KH_2_PO_4_, pH = 7.4, 1 M, [TSP] 0.01%) was centrifuged again at 3500 rpm for 5 min at 4 °C, and 600 μL of the supernatant was transferred to a 5 mm NMR tube. ^1^H NMR measurements were carried out on a Bruker Avance III HD 700 MHz spectrometer equipped with a 5-mm z-axis gradient TCI cryogenic probe (Bruker, Billerica, MA, USA). 1D ^1^H NMR spectra were acquired at 298 K with the standard NOESY-1D pulse sequence (Bruker pulse program *noesypr1d*). Water resonance was irradiated during a relaxation delay of 4.0 s and mixing time of 10 ms; 256 scans were collected into 64 K time domain complex points using a spectral width of 21 ppm, and an acquisition time of 2.3 s. An exponential line broadening factor of 0.3 Hz was applied to the free induction decay (FID) before Fourier transformation.

All ^1^H NMR spectra were automatically processed by phased-, baseline-corrected and referenced internally to the chemical shift of TSP at 0.00 ppm distortion using Chenomx NMR suite 8.3 (Chenomx Inc., Edmonton, AB, Canada) software. Then, the regions corresponding to residual water and urea peaks (4.6–4.9 and 5.5–6, respectively) were excluded. Processed spectra were normalized to the total area. It has been reported that stand-alone biomarker discovery depends on the choice of scale, i.e., the type of normalization of the NMR spectrum (total area, referred to creatinine, PQN and TSP) so that new approaches to get the best results are continuously emerging and discussed in the literature [34]. The total area normalization procedure was selected as it has been reported to give satisfactory results in longitudinal studies with urine samples of newborns using the employed analytical technique [35]. Data were exported in CSV file format and were divided into regions having an equal bin size of 0.04 ppm with ASICS, an R package available on Bioconductor (https://www.bioconductor.org/packages/rlease/bioc/html/ASICS.html accessed on December 2021).

After the significant bins were selected according to chemometrics analyses, each bin was analyzed for the presence of characteristic metabolites appearing in each one. Next, all metabolites appearing on each bin were identified and quantified. A multiple comparison t-test was then performed between the concentrations of the metabolites in the PN and EN groups. Statistical significance was determined using the Bonferroni–Dunn method. Compound identification was accomplished using the Chenomx NMR Suite software (Version 8.3, Chenomx Inc., Edmonton, AB, Canada).

### 4.5. Statistical Data Analysis

Dimension reduction techniques were applied to analyze the multiple, often correlated, measured responses. The ^1^H NMR spectra were employed for PCA and OPLS-DA to identify clinical features that could outline distinct metabolic profiles [36]. The quality of the model was determined by the goodness of fit parameter (R2) and the goodness of prediction parameter, a measure of the fraction of the total variation predicted by the model (Q2). Univariate scaling and cross validation through venetian blinds with 10 splits and blind thickness of one was employed with the aim of avoiding model overfitting due to repeat measurements on each subject. The variable importance in the projection (VIP) method and the S-line plot were used to identify metabolites responsible for the separation of nutrition groups, while the permutation plot was used to perform model assessment. The association of specific spectral data with the prematurity groups was assessed through parallel factor analysis (PARAFAC) to account for correlations from serial measurements in the same individual. All multivariate statistical data analyses were performed using PLS-Toolbox 8.7.1 software (Eigenvector Research, Inc., Manson, WA, USA), except for the S-line and permutations plots, which were performed using SIMCA software (Umetrics, Sartorius Stedim Biotech 16.0.1.7928, Umea, Sweden). Univariate statistical analyses were performed using the GraphPad Prism 8.0 (GraphPad Software, La Jolla, CA, USA).

### 4.6. Pathway Analysis

MetaboAnalyst 5.0 was used to identify the most relevant metabolic pathways that were significantly enriched in the metabolomic data. Hypergeometric tests and relative betweenness centrality were assessed. Both results were simultaneously plotted to show the most significant pathways in terms of enrichment analysis and pathway topology analysis [26].

## 5. Conclusions

The untargeted metabolomic ^1^H NMR approach performed on urine samples from preterm newborns allows us to identify the association of specific spectral data with different NTs and to understand some characteristics of the complex metabolism of this vulnerable pediatric population. Our results reveal changes in the metabolic profile related to PN treatment, with the tricarboxylic acid cycle and galactose metabolic pathways being the most impacted pathways. The low relative concentrations of citrate and succinate in the preterm newborns who received PN could indicate an energetic nutritional deficit that must be considered for better management of these patients. Further studies in a larger group of patients are needed to validate these findings.

## Figures and Tables

**Table 1:** Main causes of admission to the NICU of the studied preterm newborn population (*n* = 34 patients).

Diagnosis *	**Respiratory**	**Patient**	***n***	**1 ^+^**	**2**	**3**	**4**	**5**	**6**	**7**	**8**	**9**	**10**	**11**	**12**	**13**	**14**	**15**	**16**	**17**	**18**	**19**	**20**	**21**	**22**	**23**	**24**	**25**	**26**	**27**	**28**	**29**	**30**	**31**	**32 ^+^**	**33**	**34**
		**Respiratory** **Distress** **Syndrome**	18	♦		♦	♦		♦		♦	♦				♦	♦	♦		♦		♦		♦	♦	♦	♦								♦	♦	♦
		**Asphyxia**	5		♦	♦				♦		♦		♦																							
		**Pneumothorax**	2						♦					♦																							
		**Pulmonary** **Atelectasis**	2					♦	♦																												
		**Pulmonary** **Hemorrhage**	1				♦																														
		**Pulmonary** **Hypertension**	1	♦																																	
		**Pneumonia**	2						♦																									♦			
	**Gastrointestinal**	**Hyperbilirubinemia**	12				♦	♦		♦	♦		♦			♦	♦	♦		♦	♦								♦			♦					
		**Gastrointestinal** **Malformations**	4																♦									♦		♦			♦				
		**Necrotizing** **Enterocolitis**	2			♦							♦																								
	**Cardiological**	**Congenital Heart Defect**	8	♦			♦	♦		♦	♦	♦							♦				♦														
		**Tricuspid Valve Insufficiency**	2	♦				♦																													
		**Pericardial** **Effusion**	1			♦																															
		**Ventricular** **Tachycardia**	1		♦																																
	**Neurological**	**Hydrocephalus**	1												♦																						
		**Microcephalus**	1																												♦						
		**Intraventricular Hemorrhage**	2		♦										♦																						
		**Brain Cortex** **Atrophy**	1		♦																																

♦ = yes * Each patient could have more than one diagnosis; ^+^ Deceased patients.

**Table 2:** Metabolites and non-assigned signals in the studied preterm newborns associated with NT with VIP values > 1.5. The *p* (corr) is the absolute value of the correlation loading in the S-line plot. The *p* values refer to the two-group independent samples comparison *t* test between the PN and EN groups.

Metabolite (ppm)	HMDB ID	*p* (Corr)	*p* Value
Gluconate (3.76, 4.04, 4.16)	HMDB0000625	0.7	<0.000001
Glucose (3.28, 3.76)	HMDB0000122	0.7	0.001396
N-acetyltyrosine (1.92, 2.84, 6.84, 7.16, 7.76)	HMDB0000866	0.9	<0.000001
4-Hydroxyphenyllactate (6.84, 7.16, 4.16)	HMDB0000755	0.9	0.828728
Quinolinate (8.44)	HMDB0000232	0.4	0.236545
Succinate (2.4)	HMDB0000254	0.9	<0.000001
Galactose (4.6)	HMDB0000143	0.9	<0.000001
3-Aminoisobutyrate (2.64)	HMDB0002166	0.4	0.043601
Citrate (2.68, 2.52)	HMDB0000094	0.9	0.004906
1-Methylnicotinamide (4.48)	HMDB0000699	0.7	0.301233
Lactose (4.48)	HMDB0000186	0.7	0.000248
Myo-inositol (3.64, 4.08)	HMDB0000211	0.7	0.859912
Betaine (3.28)	HMDB0000043	0.4	0.100100
N,N-dimethylglycine (2.52, 2.92)	HMDB0000092	0.4	0.702205

**Table 3:** Buckets´ chemical shifts associated with the absolute difference between the mode 2 factors of the PARAFAC-2 model and their corresponding metabolites in the studied preterm newborns.

Buckets/ppm	Absolute Difference	Metabolites
3.72–3.68	0.4319149	Non-assigned signals
3.28–3.24	0.1895293	Betaine, Glucose
3.56–3.52	0.1583987	Myo-inositol, Glycine
3.24–3.20	0.1501642	Glucose
3.84–3.80	0.096618	Gluconate, Glucose
3.92–3.88	0.0817509	Non-assigned signals
3.04–3.00	0.076888	Creatinine
3.96–3.92	0.0752876	Non-assigned signals
4.08–4.04	0.0723879	Myo-inositol, Creatinine
3.60–3.56	0.0700471	Myo-inositol
2.08–2.04	0.0690634	Non-assigned signals
4.00–3.96	0.0658843	Non-assigned signals
3.52–3.48	0.0632958	Myo-inositol
3.64–3.60	0.0605931	Myo-inositol
2.04–2.00	0.0518613	Non-assigned signals
4.16–4.12	0.0470665	Gluconate, 4-Hydroxyphenyllactate
2.40–2.36	0.0461973	Succinate

## Data Availability

The data that support the findings of this study are available upon reasonable request [The data are not publicly available due to privacy/ethical considerations].

## Supplementary Materials

The following supporting information can be downloaded at: https://www.mdpi.com/article/10.3390/metabo12030255/s1, Figure S1: Permutation test conducted with 300 randomly initiated permutations showing R2 (green circles) and Q2 (blue squares) values from the permuted analysis (left-bottom corner) and the associated OPLS-DA model values (right-top corner). Intercepts: R2 = (0.0, 0.318), Q2 = (0.0, −0.374); Figure S2: S-line plot showing the predictive loadings (p(ctr) [1]) as a function of the original variables (ppm) colored according to the absolute value of the correlation loadings (p(corr) [1]); Figure S3: Core consistency plot of the PARAFAC-2 model; Figure S4: Loading plot (loadings vs. time) of the first mode (time) in the PARAFAC-2 model; Figure S5: Loading plot (loadings vs. chemical shifts) of the second mode (spectra) in the PARAFAC-2 model; Table S1. Detailed results from the pathway analysis; Table S2. ANOVA results of comorbidities for each NT group Table S3. Characteristics of the PN used in the studied preterm newborns; Table S4. Characteristics of the EN used in the studied preterm newborns.
