# Supplementary material for: A Longitudinal 1H NMR-Based Metabolic Profile Analysis of Urine from Hospitalized Premature Newborns Receiving Enteral and Parenteral Nutrition
Source: Metabolites. 2022 Mar 17;12(3):255. doi: 10.3390/metabo12030255 (PMC8952338; doi:10.3390/metabo12030255)
Supplement: Supplementary file 1 [file metabolites-12-00255-s001.zip › metabolites-1610475-Supplementary.pdf]

metabolites

MDPI

Article

A Longitudinal ^{1}H NMR-Based Metabolic Profile Analysis of Urine from Hospitalized Premature Newborns Receiving Enteral and Parenteral Nutrition

Nuria Esturau-Escofet [1,†], Eduardo Rodríguez de San Miguel [2,†], Marcela Vela-Amieva [3,†], Martha E. García-Aguilera [1], Circe C. Hernández-Espino [1], Luis Macias-Kauffer [4], Carlos López-Candiani [5], José J. Naveja [1] and Isabel Ibarra-González [6,*]

Supplementary material

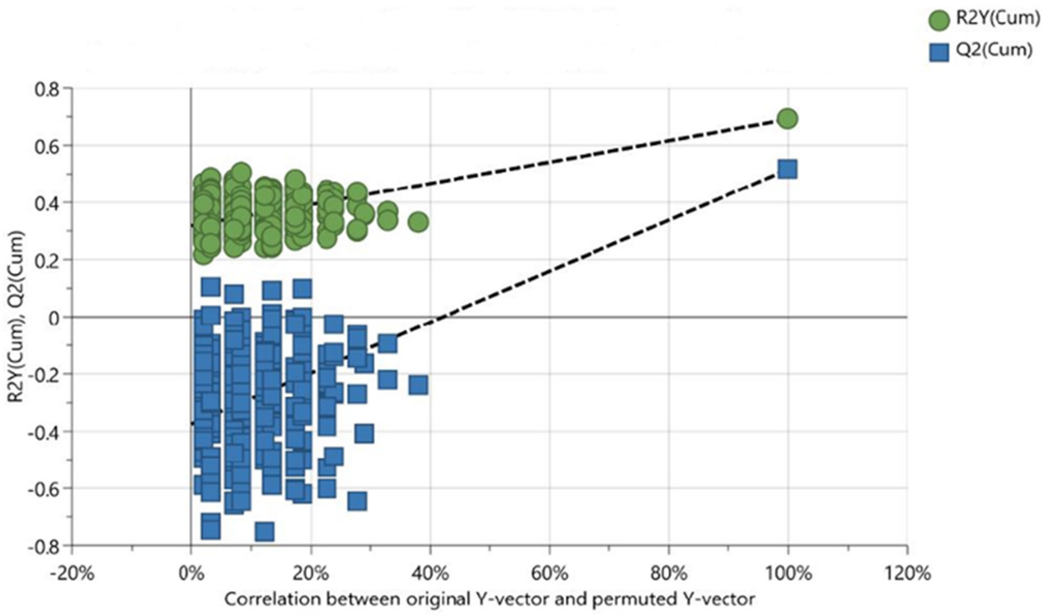

Supplementary Figure S1. Permutation test conducted with 300 randomly initiated permutations showing R2 (green circles) and Q2 (blue squares) values from the permuted analysis (left-bottom corner) and the associated PLS-DA model values (right-top corner). Intercepts: R2 = (0.0, 0.318), Q2 = (0.0, -0.374).

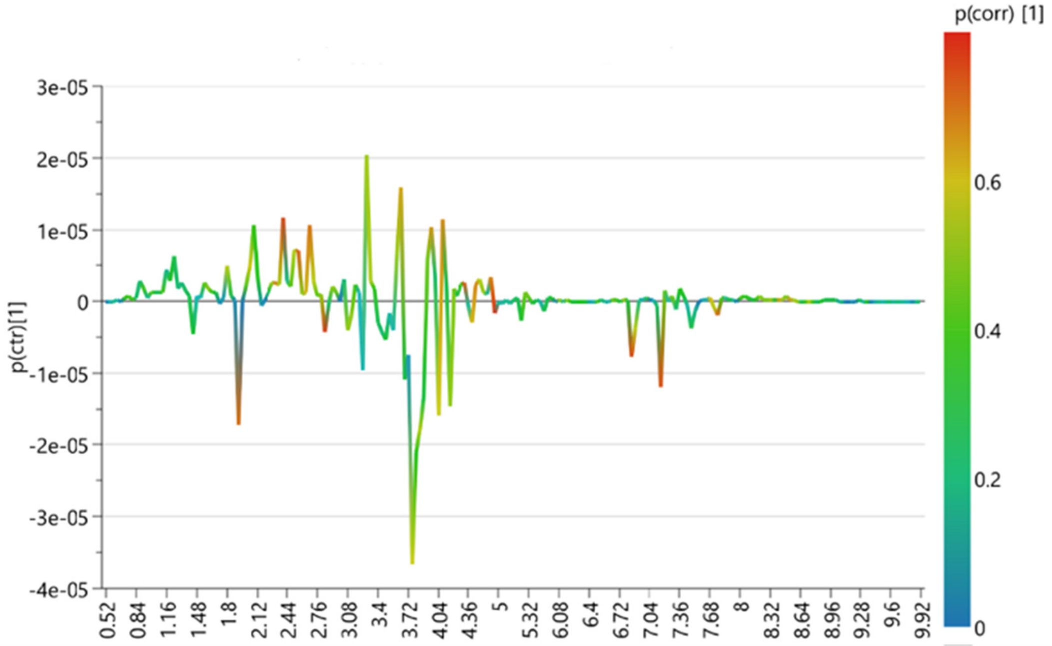

Supplementary Figure S2. S-line plot showing the predictive loadings (p(ctr)[1]) as a function of the original variables (ppm) colored according to the absolute value of the correlation loadings (p(corr)[1]).

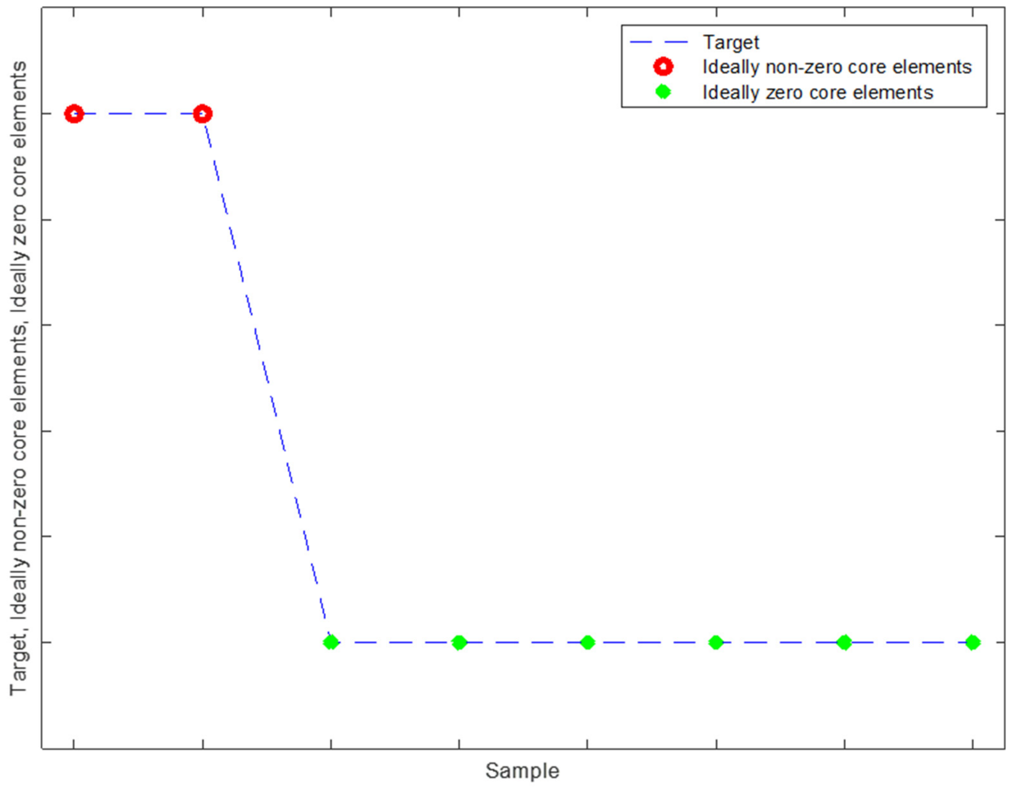

Supplementary Figure S3. Core consistency plot of the PARAFAC-2 model.

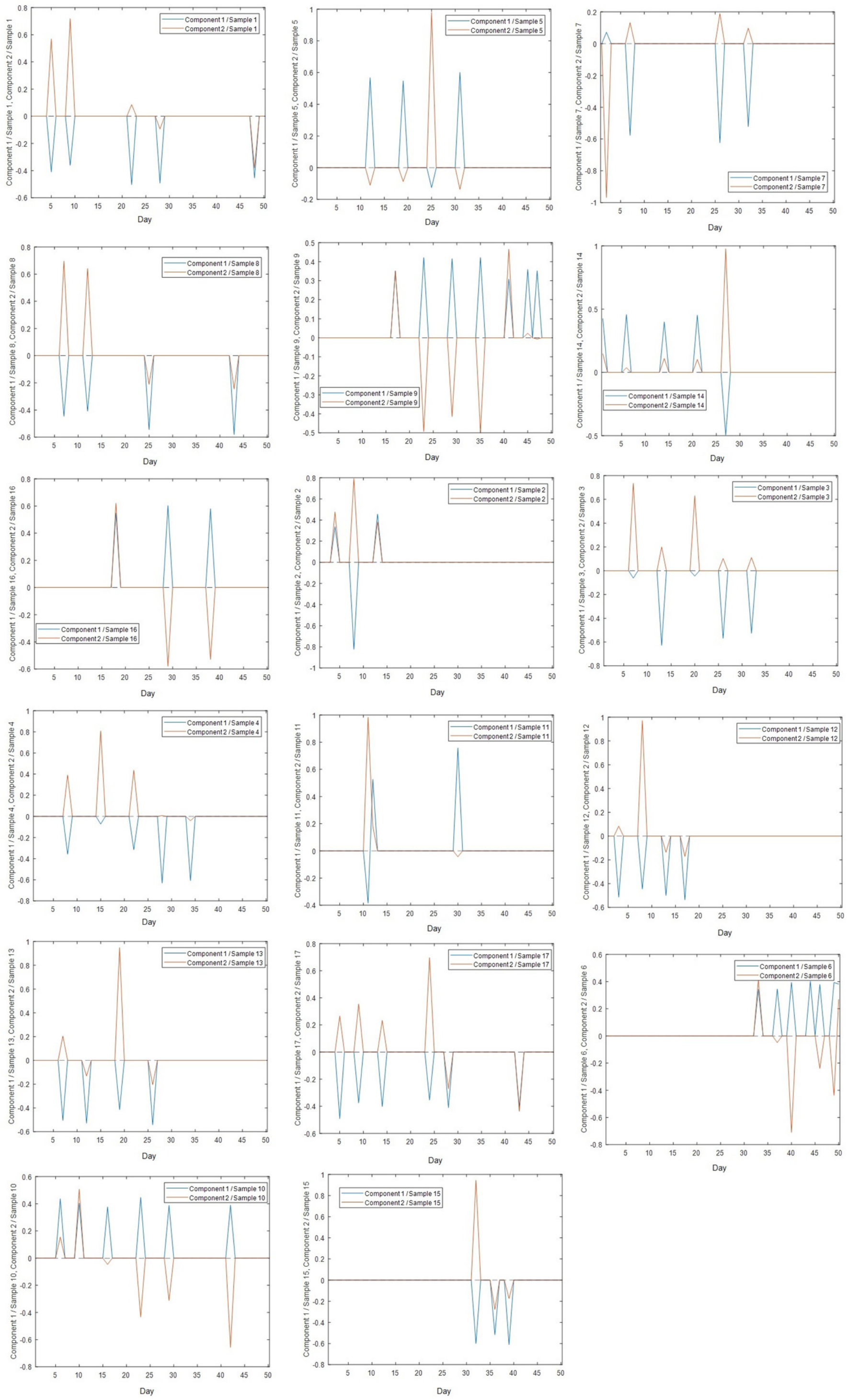

Supplementary Figure S4. Loading plot (loadings vs. time) of the first mode (time) in the PARAFAC-2 model. Two factors (blue and orange) are represented among 17 subjects.

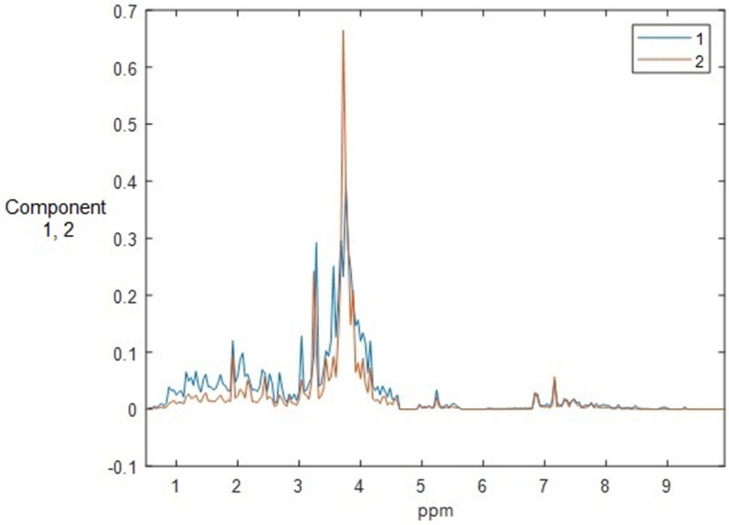

Supplementary Figure S5. Loading plot (loadings vs. chemical shifts) of the second mode (spectra) in the PARAFAC-2 model. Two factors (blue and orange) are represented according to their related chemical shifts.

Supplementary Table S1. Detailed results from the pathway analysis.

Pathway Name	Total	Hits	p	-log (p)	Impact
Galactose metabolism	27	2	0.009760	2.011	0.1632
Citrate cycle (TCA cycle)	20	2	0.005396	2.268	0.1231
Pentose phosphate pathway	22	1	0.121020	0.917	0.0471
Glyoxylate and dicarboxylate metabolism	32	1	0.171590	0.766	0.0318
Glycolysis/Gluconeogenesis	26	1	0.141570	0.849	0.0002
Alanine, aspartate, and glutamate metabolism	28	2	0.010479	1.980	0
Butanoate metabolism	15	1	0.084009	1.076	0
Propanoate metabolism	23	1	0.126200	0.899	0
Amino sugar and nucleotide sugar metabolism	37	1	0.195890	0.708	0
Valine, leucine, and isoleucine degradation	40	1	0.210160	0.677	0

Total is the number of all compounds in the pathway; Hits is the matched number of metabolites from the uploaded data; p is the original p value calculated from enrichment analysis; and the impact value is calculated from topology analysis.

Supplementary Table S2. ANOVA results of comorbidities for each NT group.

	SS	DF	MS	F (DFn, DFd)	P value
Comorbidities Factor	2370	17	139.4	F (17, 17) = 3.919	P=0.0037
NT Factor	2.25×10^{-16}	1	2.25×10^{-16}	F (1, 17) = 6.325×10^{-18}	P>0.9999
Residual	604.8	17	35.58		

Supplementary Table S3. Characteristics of the PN used in the studied preterm newborns.

Proteins<br>1 g= 4 kcal<br>Doses: 4 g/kg	Pediatric amino acids 10%, LE-VAMIN® PAD, Pisa® Farmaceutica, Mexico.	Each 100 ml contains:<br>Essential amino acids:<br>L-Isoleucine 820.0 mg; L-Leucine 1400.0 mg; L-Lysine 850.0 mg; L-Methionine 340.0 mg; L-Phenylalanine 480.0 mg; L-Threonine 420.0 mg; L-Tryptophan 200.0 mg; L-Valine 780.0 mg; L-Cysteine 16.0 mg; L-Histidine 480.0 mg; L-Tyrosine 240.0 mg.<br>Nonessential amino acids:<br>L-Alanine 540.0 mg; L-Arginine 1200.0 mg; L-Proline 680.0 mg; L-Serine 380.0 mg; Glycine 360.0 mg; L-Aspartic Acid 320.0 mg; L-Glutamic Acid 500.0 mg<br>L-Taurine 25.0 mg; Sodium metabisulfite (preservative) < 50.0 mg.<br>Approximate number of electrolytes:<br>Sodium 5 mEq/L<br>Chloride < 3 mEq/L<br>Acetate 97 mEq/L<br>Osmolarity: 909 mOsmol/L
Carbohydrates<br>1 g= 3.44 kcal<br>Doses: 17.2 g/kg	Glucose anhydrous 50% SOLUCION DX-50 PISA® Farmacéutica, Mexico.	Each 100 ml contains:<br>Glucose 50 g<br>Water for injection 100 ml.
Lipids<br>1 g= 10 kcal<br>Doses: 3.5 g/kg	Medium and long-chain fatty acids, LIPOFUNDIN ®MCT/LCT, PISA® Farmacéutica, Mexico	Each 100 ml contains:<br>Soybean oil 10.0 g; Medium chain triglycerides (MCT) 10.0 g.<br>Phosphate equivalent: 14.5 mmol/L<br>Caloric content: 7990 (1908) kj/L (kcal/L)<br>Approximate osmolarity: 380 mOsm/L
	Soybean oil, olive, fish oil, oil, medium chain triglycerides, SMOF-lipid®, Fresenius Kabi Mexico.	Every 100 ml contains<br>Refined soybean oil 6.0 g; Medium chain triglycerides 6.0 g; Refined olive oil 5.0 g; Fish oil rich in omega-3 acids 3.0 g.<br>Total energy input: 8.4 MJ/l (= 2000 Kcal/l)<br>Osmolality approx: 380 mOsmol/kg.
Multivitamin<br>3 ml	Pediatric Multivitamin, VITA-FUSIN®, PISA® Farmacéutica, Mexico.	1 ampoule of 5 ml contains<br>Vitamin C: 80 mg; Folic acid: 0.140 mg; Biotin: 0.020 mg; Vitamin B12: 0.001 mg; Pantothenic acid: 5.0 mg; Vitamin B2: 1.4 mg; Niacinamide: 17 mg; Vitamin B6: 1.0 mg; Vitamin B1: 1.2 mg; Retinol or Vitamin A: 2000 IU; Vitamin D3: 200 IU; Vitamin E: 7 IU; Vitamin K1: 0.2 mg.
Trace elements<br>0.3 ml/kg	Tracefusin®, PISA® Farmacéutica, Mexico.	Every 100 ml contains: Zinc chloride 55.00 mg; Cupric sulphate pentahydrate 16.90 mg; Manganese sulphate 38.10 mg; Sodium iodide 1.30 mg; Sodium fluoride 14.00 mg; Sodium chloride 163.90 mg.
Glutamine<br>0.3 g/kg	Alanine and Glutamine, DIPEPTIVEN®, Fresenius Kabi Mexico.	Each 100 ml contains: N(2)-L-alanyl-L-glutamine (equivalent to 8.20 g of L-alanine, and 13.46 g of L-glutamine) 20.0 g.<br>Theoretical osmolarity 921 mOsmol/l
Carnitine<br>5 mg	EFE-CARN®, PISA® Farmacéutica, Mexico.	Each ampoule 5 ml contains: 1 g L-carnitine.
Zinc<br>100 microg/kg	ZN-FUSIN®, PISA® Farmacéutica, Mexico.	Each ml contains: Zinc sulphate heptahydrate, equivalent to 1 mg of elemental zinc.

Supplementary Table S4. Characteristics of the EN used in the studied preterm newborns.

Enfamil Prematuros Premium®, Mead Johnson®, Mexico	Per 100 kcal	Per 100 g, power	24 kcal/30 ml (81 kcal/100 ml)
Energy, KJ (kcal)	427 (100)	2050 (490)	343 (81)
Protein, g	3	14.7	2.4
Lipids, g	5.1	25	4.1
Carbohydrates, g	11	54	8.9
Vitamin A, µg	150	750	123
Vitamin D, µg	2.5	12.3	3.4
Vitamin E, µg	4.2	21	3.4
Vitamin K, µg	8	39	6.5
Thiamine, µg	200	980	162
Riboflavin, µg	300	1470	240
Vitamin B6, µg	150	740	122
Vitamin B12, µg	0.25	1.2	2
Niacin, µg	4000	19600	3200
Folic acid, µg	40	196	32
Pantothenic acid, µg	1200	5900	970
Biotin, µg	4	20	3.2
Vitamin C, mg	20	98	16.2
Choline, mg	18	88	14.6
Inositol, mg	45	220	36
Taurine, mg	6	29	4.9
Carnitine, mg	2	9.8	1.62
Calcium, mg	120	590	97
Phosphorus, mg	66	320	53
Magnesium, mg	9	44	7.3
Iron, mg	0.5	2.4	0.4
Zinc, mg	1	4.9	0.81
Manganese, µg	6.3	31	5.1
Copper, µg	120	590	97
Iodine, µg	25	123	20
Sodium, mg	58	284	47
Potassium, mg	100	490	81
Chloride, mg	85	420	69
Linoleic acid, mg	810	4000	660
Linolenic acid, mg	110	540	89
Docosahexaenoic acid, mg	17	83	13.8
Arachidonic acid, mg	34	167	28
Selenium, µg	1.1	5.4	0.9
